# Supplementary material for: Chromothripsis during telomere crisis is independent of NHEJ, and consistent with a replicative origin
Source: Genome Res. 2019 May;29(5):737–49. doi: 10.1101/gr.240705.118 (PMC6499312; doi:10.1101/gr.240705.118)
Supplement: Supplemental Material [file supp_gr.240705.118_Supplemental_file_1.zip › contigs/annotated_contigs/DB77/contig.2.DB77_length_545_mean_cov_6.60550458716.docx]

**DB77_length_545_mean_cov_6.60550458716**

```
CATGCACGTAGCAAAAACACCAGGGGTCTTGAGAGGTCATAGCCTGAAAAGGAAGTAGAGAGAGAAGCAGAGGAAGTGACACTTAACGT
 >chr1:28211643-28211960 + E=4e-176
GAATGACCGTTTTTCATGGACTCTGCTTCCCCATTTCTTGAGACCCTGATGATTTCTGCTTTTTCTCCCAAAGTCTTCTCAAGTCTTAG

GATTGCAGCAACACGCTCGGCTGCCCAAACCCAAGGCGAAGACCTTGCCAGAGTTCATCAAGGATGGCTCCAGTACGTACAGCAAGATT

CCTGCCCACAGGAAGGGCCACAGGCCCGCTAAGCCCCAAAGGCAGGATCT|GAT|AAGCCCCATGTGAAGCAAAGGAGAGGTACTCCCA
                                                      >chr1:28276702-28276927 - E=9e-12
GTTGGATCTGAGTTTGAGTCCCTCCCTCGGAACCGACTAGCTCGGAGCCACAGGTTCCTTTTCTATACATTGGGAATAATGGTGCCTGC
4
TGTTGGGGTCAGGGGAATGTCCTGGTTTGACCCCTGCTGGAAGACAAAGGAAACCAAGCAGCCTGACTTGGGTTGGCTGGGTTAGATAA

CCTGGAAGCATCC
```
